# Thermal Restriction as an Antimicrobial Function of Fever

**DOI:** 10.1371/journal.ppat.1005577

**Published:** 2016-05-05

**Authors:** Arturo Casadevall

**Affiliations:** Department of Molecular Microbiology and Immunology of the Johns Hopkins School of Public Health, Johns Hopkins University, Baltimore, Maryland, United States of America; Geisel School of Medicine at Dartmouth, UNITED STATES

*Calor* (heat), *rubor* (redness), *tumor* (swelling), and *dolor* (pain) are the cardinal signs of inflammation. To these can be added loss of function by inflamed tissues. Of the cardinal signs, *calor*, or fever, is a common response to infection. Fever is an evolutionarily conserved host response to microbial infection that has been described in organisms from such diverse phyla as Chordata, Arthropoda, and Annelida [1]. Fever has been defined as “a state of elevated core temperature, which is often, but not necessarily, part of the defensive responses of multicellular organisms (host) to invasion of live (microorganisms) or inanimate matter recognized as pathogenic or alien to the host” [2]. It is noteworthy that this definition views fever as an increased temperature, and in this essay we will focus on the effects of temperature on host defense. For endothermic and homoeothermic organisms like mammals, fever reflects an increase in metabolism such that maintaining a fever temperature 2°C above the afebrile state can increase metabolism by 20% [3]. For ectothermic organisms, fever can result from increased metabolic activity and/or be induced by increased physical activity or exposure to warm sources like the sun. In insects and ectothermic invertebrates, there is a large body of evidence showing that fever is beneficial to the host during microbial infection [3]. However, in mammals, some have questioned whether this increase in metabolism over an already high baseline is beneficial and suggested that the risks and discomfort associated with increased temperature outweigh any benefits [4], a view that also considers fever as an unpleasant side effect of the response to infection. An unequivocal demonstration of the benefits of fever in mammals has proved elusive, with some studies supporting a beneficial role for fever, while others note detrimental effects [1,5,6]. While there is no question that life-threatening elevations in temperature require antipyretic treatment, there is much less consensus on how to approach common fevers. Perhaps the best evidence for a dismissive approach to the value of fever in humans is the fact that physicians, nurses, and parents reflexively respond to fever by administering antipyretics [5,7,8].

If fever is an evolutionarily conserved response to microbial infection in metazoan organisms, which presumably reflects an adaptive response, then why has it been so difficult to unequivocally demonstrate a beneficial role in mammals and particularly in humans? There are several factors that make answering this question experimentally difficult [9]. Any change in host temperature will simultaneously affect several variables. Temperature has separate effects on the immune system and on the microbe and may affect their interaction such that host–microbe interactions differ at different temperatures. Mammals already have elevated temperatures, and the only mechanism for raising their temperature involves inducing artificial fevers that can have a variety of non-physiological effects. Similarly, reducing fever using antipyretics eliminates a physiological response, and some, like aspirin, have effects on prostaglandin synthesis such that it is difficult to conclude that the effect is due to temperature alone. The fact that it is not possible to isolate one variable, vary it, and measure an outcome means that the problem of the contribution of fever to host defense is innately resistant to reductionist experimental approaches. In fairness to all the efforts that have been made, there is a large body of evidence correlating fever with enhanced resistance to microbial diseases, but what is missing, at least in mammals, is an unambiguous demonstration that this relationship is causative.

Despite the difficulties in teasing out the experimental variables, it is possible to reduce the problem of fever to a metabolic state that generates a host that is warmer and potentially more thermally inhospitable to certain microbes. All microbes have an upper temperature limit at which replication and viability are impaired, which is termed the maximum tolerated temperature. Although replication and viability are different parameters, impairment of either, or both, during infection can be expected to translate into a benefit for the host by limiting the number of microbes. If the host responds to infection with a fever that reaches or exceeds the maximum tolerated temperature of the microbe, then fever is unequivocally important in providing a thermal exclusionary zone against that specific microbe. It is noteworthy that this principle was used therapeutically in the pre-antibiotic era. In 1917, Julius Wagner-Jauregg began to experiment with inducing fevers for treating convulsive diseases based on the administration of live *Plasmodia* and was awarded the Nobel Prize in 1927 [10]. Fever therapy was applied to infectious diseases and by the 1930s was routinely used for the treatment of syphilis and gonorrhea [11]. Malaria-induced fever therapy for syphilis resulted in full remission in 30% of cases, and for those that relapsed, retreatment was recommended [12]. For both syphilis and gonorrhea, the thermal tolerance of the causative microbes was lower than the induced fevers, which created a thermal restriction zone that translated into a therapeutic effect [11]. With time, malaria therapy gave way to artificial fevers induced by placing the patient in a chamber known as the Kettering Isotherm, where a fever of 40.6°C could be achieved in less than one hour, and the process had few side effects [11]. Although the results from these early studies have to be approached cautiously, since they were not controlled by current standards and artificial hyperthermia could also have stimulated innate immunity, they are supportive of the notion that heat can mediate a therapeutic effect by directly inhibiting the microbe. Fever therapy was abandoned with the introduction of effective antimicrobial therapy, but that chapter of medical history remains informative for the therapeutic potential of higher temperatures and is potentially relevant to the discussion of the role of fever in host defense.

In recent years, the ability of higher temperatures to cure certain diseases has been demonstrated in amphibians, mammals, and fish. Frog and bat populations have each been decimated by fungal species that cannot tolerate high temperatures. Frogs infected with the chytrid *Batrachochytrium dendrobatidis* and bats infected with *Pseudogymnoascus destructans* can each be cured if their body temperatures are increased to temperatures that are not tolerated by the fungus [13,14]. In the case of frogs, this is accomplished by placing them in a warm room. For bats, susceptibility to *P*. *destructans* is associated with hibernation, when their body temperatures are 10–12°C, and simply feeding them such that they can maintain a normal temperature is sufficient to clear the infection [14]. Guppies infected with the parasite *Gyrodactylus turnbulli* cured themselves of infection by purposely selecting warmer waters that inhibited parasite growth [15]. In each of these cases, cure was mediated by host survival in temperatures that were not tolerant to the microbe, which effectively created a thermal restriction zone.

If fever is considered as a mechanism for creating a thermal restriction zone in the host, then its protective properties against thermally intolerant microbes are immediately apparent. An analysis of the tolerance of fungal strains showed that in the region of 30–44°C, a one-degree increase in temperature was associated with inhibition of approximately 6% of fungal species, with the cumulative effect being such that the majority of fungal species cannot survive in mammalian temperatures [16]. Although comparable data on bacterial species is not available, the available evidence suggests that the majority of environmental bacteria would also find mammalian hosts a thermally restricted environment. For example, a survey of the thermal tolerances of marine bacteria revealed that the majority were inhibited by a temperature of 25°C, which is considerably cooler than mammalian temperatures [17]. It is noteworthy that mammals can differ significantly in their basal temperatures [18], and some, like rabbits, have basal temperatures of 39–40°C, which are thought to contribute to their high resistance to experimental fungal infection with *Cryptococcus neoformans* [19].

Focusing on fever as a mechanism for creating a thermally restrictive host also suggests another explanation for why it has been difficult to unequivocally establish a role for fever in mammalian host defense. In this regard, the majority of experimental work has focused on viruses and bacteria that are known mammalian pathogens and are thus already adapted to higher temperatures. For these microorganisms, the increase in temperature is not sufficient to create a thermally restrictive zone. For example, one of the most convincing experimental models suggesting a protective role for fever used intrathecal *Streptococcus pneumoniae* infection in mammals and demonstrated a reduction in growth, but there was still bacterial replication at the higher temperature, and this experiment could not control for effects of the drugs on host immunity independent of thermal effects [20]. Hence, the efficacy of fever as a defense mechanism may vary with the pathogenic microbe, and if that is the case, protective effects may be diluted away when the data are analyzed in aggregate.

In summary, viewing fever as a host response that increases its thermal restriction provides another way to approach the contribution of fever to host defense. The concept of thermal restriction is obvious but does not seem to have taken a hold in the zeitgeist, probably because investigators are more focused on thermal effects on the immune system. In this regard, fever has protean effects on the immune system that in aggregate are likely to help the host control infection [21,22]. The secretion of pyrogenic cytokines in response to infection can enhance local immune responses, and the resultant fever responses are associated with enhanced neutrophil oxidative burst and migration into infected tissues [21]. Most infectious diseases today are acquired from other mammalian hosts, and thus the microbe arrives at the new host already acclimated to the temperature span included by a fever response. However, this was probably not always the case in the evolution of our species. Solitary mammals and those that live in small groups may have faced a greater threat from environmental microbes than from other mammal-adapted microbes, and if this was the case, the higher thermal restriction caused by a fever response would have provided considerable protection. Perhaps it is time to re-evaluate the beneficial effects of fever in light of the thermal tolerance of the offending microbe. The concept of host thermal restriction is also important as we explore space and come to appreciate concerns about microbial threats from exobiology. For example, any microbial life on Mars is almost certainly cold-adapted relative to life on balmy earth and, as such, is unlikely to pose a threat to mammals. On the other hand, any Venusian microbial life would be hot-adapted and presumably could pose a greater threat to mammals, as the thermal restriction would not be sufficient to inhibit it. Viewed from the prism of endothermy and fever, warmer worlds containing microbial life are potentially more dangerous than colder worlds.
